# A tailored smoking cessation intervention for people with multiple sclerosis: Results of a single-arm feasibility study

**DOI:** 10.1177/27558428261444225

**Published:** 2026-05-16

**Authors:** Alex M. Keller, Daniel Kotz, Claudia H. Marck, Martin Winter, Chris M. Friemel, Christoph Heesen, Karin Riemann-Lorenz

**Affiliations:** 1 Institute of Neuroimmunology and Multiple Sclerosis, University Medical Centre Hamburg-Eppendorf, Germany; 2 Institute of General Practice, Addiction Research and Clinical Epidemiology Unit, Centre for Health and Society, Medical Faculty and University Hospital Düsseldorf, Heinrich Heine University Düsseldorf, Germany; 3 Disability and Health Unit, Centre for Health Policy, The Melbourne School of Population and Global Health, The University of Melbourne, VIC, Australia; 4 Institute for Occupational and Maritime Medicine, University Medical Centre Hamburg-Eppendorf, Germany; 5 IFT Mental Health Solutions GmbH, Munich, Germany

**Keywords:** multiple sclerosis, smoking cessation, intervention, feasibility, acceptability

## Abstract

**Background:**

People with multiple sclerosis (pwMS) who smoke have an increased risk of progression. MS-specific barriers make it more difficult to stop smoking. However, tailored smoking cessation programs for pwMS are non-existent.

**Objective:**

The aim of this study was to assess the feasibility of a newly developed MS-specific smoking cessation intervention.

**Design:**

This was a one-armed, uncontrolled, prospective feasibility study. The intervention consisted of five online group sessions over 3 weeks, with follow-up assessments 3 and 12 weeks after baseline. This study took place between December 2024 and September 2025.

**Methods:**

Recruitment was conducted via clinics and MS networks. Primary outcomes were demand, practicality, and acceptability. The potential effectiveness was a secondary outcome. Self-reported smoking status was assessed at both follow-ups. Smoking status for participants who were smoke-free at 12 weeks was verified by measuring cotinine levels in saliva samples. This study was pre-registered: https://drks.de/search/de/trial/DRKS00035557.

**Results:**

With regard to demand, we screened 130 individuals from November 2024 to May 2025, with 99 found to be eligible. Of those enrolled (*n* = 62), 48 started the intervention in 8 groups of 4–10 participants. With regard to practicality, 83% (*n* = 40) completed the 3-week follow-up, and 77% (*n* = 37) the 12-week follow-up. Acceptability was high, with all participants who completed the questionnaires (*n* = 39) stating that they would recommend the program. Respectively, 48% (*n* = 23) and 25% (*n* = 12) of participants self-reported to be smoke-free at 3 and 12 weeks. We did not reach the target sample size of 60, and the dropout rate at 12 weeks (*n* = 11, 23%) slightly exceeded our predefined 20% threshold.

**Conclusion:**

The intervention was well received. A randomized controlled trial to test its efficacy seems feasible, with improvements to recruitment strategies and by integrating participants’ feedback. Our findings suggest that tailored interventions may play an important role in addressing smoking cessation in people with other chronic or neurological conditions as well.

**Trial registration:**

This study was registered at the German Clinical Trials Register: DRKS00035557.

## Plain language summary

Can an online group program help people with multiple sclerosis quit smoking?

Why was the study done?

Smoking can make the symptoms of multiple sclerosis (MS) worse and speed up the progression of the disease. However, many people with MS find it difficult to quit. We wanted to see if a specially designed online support program could help and if a larger, future study of this program is worthwhile.

What did the researchers do?

We tested an online program consisting of five group meetings over three weeks. 48 people with MS who wanted to quit smoking took part. We used questionnaires to ask them about their experiences immediately after the program and again 12 weeks later to see if they were still smoke-free.

What were the main findings?

The program was very well received. 39 participants said they would recommend it to others. Three weeks after starting, 23 people had stopped smoking. 12 weeks after starting, 12 people remained smoke-free.

What do these results mean?

Because so many people stayed in the study and gave positive feedback, we found that this type of support is a practical way to help people with MS to quit smoking. These results justify conducting a larger study to prove exactly how effective the program is in helping people with MS quit.

## Background

Multiple sclerosis (MS) is a chronic neurological condition that manifests due to genetic vulnerability coupled with environmental factors,^1^ leading to substantial disability in at least two-thirds of people with MS (pwMS). Recent studies have highlighted tobacco smoking as a relevant determinant of MS onset.^2^ A 2017 meta-analysis of eight datasets showed that people who smoked had an increased hazard ratio of 1.55 (95% confidence interval (CI): 1.1–2.2) for faster progression of MS compared to non-smokers.^3^ Smoking is also associated with lower quality of life,^4^ increased depression and anxiety,^5^ worse long-term cognitive function,^6^ and higher premature mortality^7^ among pwMS. On the contrary, current evidence indicates that stopping smoking can have significant benefits for pwMS. Persistent smokers reach the progressive stage of MS earlier compared with those who stop at time of diagnosis.^8^ Smoking cessation can reduce the risk of reaching disability milestones, and the earlier people with MS or clinically isolated syndrome stop, the stronger this effect.^9^ However, to our knowledge, no smoking cessation programs have been designed and evaluated in MS populations.^10^ Hence, Marck et al.,^11^ in 2020, stated that research was urgently needed to maximize smoking cessation success and improve health outcomes among pwMS.

In Germany, 28% of the population older than 14 years smoke, which is one of the highest rates in Western European countries.^12^ While exact smoking rates among German pwMS are unknown, different studies indicate prevalence rates similar to the general population: Goldin et al.^13^ reported that 24.4% of a German clinical cohort of 399 pwMS were identified as current smokers. This prevalence was based on self-reported data categorizing individuals as never-smokers, current smokers, or ex-smokers. Also, Lutfullin et al.,^14^ in a nationwide German National MS cohort of 1066 newly diagnosed patients, found that 31.6% reported smoking at baseline.

Previous studies from our research group^15^ and from Australia^16,17^ have identified that receiving an MS diagnosis and related health concerns are relevant motivators to stop smoking among pwMS. Conversely, concerns about potential negative (health) consequences of smoking cessation were identified as important barriers. For instance, some pwMS reported concerns that the stress of nicotine withdrawal might lead to a MS relapse. Further, pwMS explained that they seldomly received quitting advice from their neurologist or practitioner, expressing a desire for better communication with MS clinicians and expert-led cessation interventions. Furthermore, both our and the Australian study highlighted that pwMS who smoked often had little knowledge about the effects of smoking on faster MS progression. Moreover, a substantial need for cessation services was reported in a report commissioned by the UK MS society^18^ in 2018.

To address the existing research gap and improve smoking cessation rates in pwMS, we developed an MS-specific smoking cessation intervention. The development process was systematically guided by the Behaviour Change Wheel and the COM-B Model,^19,20^ which allowed us to identify the specific capabilities, opportunities, and motivations (COM) required for behavior (B) change in the given population. This ensured that the intervention specifically targets the aforementioned unique barriers faced by pwMS. The MS-specific content included, for example, explanations on how cigarette smoke affects the central nervous system; a practical comparison of MS relapse symptoms and nicotine withdrawal symptoms; or experience reports of other pwMS who had successfully quit in the past. Detailed information on the development, the theoretical framework, and the initial testing of the MS-specific components of the intervention has been published elsewhere.^21^ The aim of the current study was to test the whole intervention feasibility, and potential effectiveness; whether the intervention shows promise of achieving the intended effects on key outcomes within a controlled setting.^22^ The feasibility framework from Bowen et al.^22^ suggests key areas of focus and possible outcomes for feasibility studies. We identified those most relevant in our context and formulated our research questions based on them^22^:

1. Demand: How much interest in, and actual use of, the program exists among pwMS?
2. Practicality: To what extent can the program be carried out by pwMS?
3. Acceptability: To what extent is the program acceptable for pwMS?
4. Potential effectiveness: Does the program indicate to be successful in terms of smoking cessation rates?

## Methods

The reporting of this study adheres to the CONSORT 2010 extension for pilot and feasibility trials checklist.^23^

### Study design

This is a one-armed, uncontrolled feasibility study of an MS-specific smoking cessation intervention to estimate whether a comprehensive effectiveness study is justified. This study was pre-registered (German Clinical Trials Register: https://drks.de/search/de/trial/DRKS00035557) and a study protocol has been made available online (https://osf.io/etvqh) prior to the start of the study. This study took place between December 2024 and September 2025.

### Participants

Participants with a self-reported MS diagnosis, aged 18 or above, who are current smokers of tobacco cigarettes, and who signed informed consent were eligible. PwMS who were not able to provide informed consent were excluded. We aimed to recruit 60 pwMS. With a sample size of 60 participants, we can determine a two-sided 95% confidence interval with a width of 0.175 when the sample proportion of non-smokers is around 0.11^24^ at the measurement after 12 weeks. Recruitment took place in the MS Outpatient Clinic of the University Medical Centre Hamburg-Eppendorf (UKE), Hamburg, Germany, where neurologists approached pwMS personally. Additionally, we used flyers and posters at our facility and sent them to other MS day clinics and neurologists across Germany who agreed to help distribute the material. We also asked MS societies in Germany to distribute study information online or via email newsletters. PwMS interested to participate completed our screening questionnaire via the data-protected online survey tool LimeSurvey. If eligible, they gained access to our study platform “eRECOVER” where they received the study patient information, were able to join the online meetings of the intervention, found complementary MS-specific information material, and took part in the baseline and follow-up assessments.

### Intervention

The intervention description adheres to the TIDieR checklist: the template for intervention descriptions and replication.^25^

The main aim of the intervention was to help pwMS to stop smoking. PwMS face different barriers and experience different motivators relevant to successful smoking cessation compared to the general population.^15–17^ Based on these findings, we developed an online smoking cessation intervention specifically for pwMS in collaboration with the Institute for Therapy Research (IFT, Munich, Germany) and the University of Melbourne, Australia (A/Prof. C.H. Marck). The program is based on an existing evaluated program of the IFT (“Das Rauchfrei Programm”/“The Smokefree Program”) for the general population, which is based on cognitive behavioral therapy and includes a variety of behavior change techniques, for example, cognitive methods to influence attitudes and motivation, or educational methods to convey an understanding of the unhealthy behavior.

The study intervention included five online group sessions for all participants (approximately 90 min each) over a period of 3 weeks. Each group consisted of up to 10 participants and was led by IFT-certified trainers with a background in psychology, pedagogy, or similar. Between the third and fourth sessions, all participants received a 10-min, one-on-one phone conversation with the trainer. Participants had access to the aforementioned online platform “eRecover,” which provided MS-specific content discussed during the sessions. For the non-MS-specific content, participants received a participant handbook prior to the first session, which guided them through the material covered during the five sessions. Surveys were conducted online: at baseline (before the first session), after the last session (3 weeks after baseline), and 12 weeks after baseline. Participants who reported being smoke-free after 12 weeks received a saliva sample kit to biochemically verify smoking cessation.

### Outcomes

Table 1 provides an overview of primary and secondary outcomes, covariates, and at which time points outcomes were assessed. To assess demand and practicality, we collected detailed descriptive data on the participant flow and recruitment for each stage of the study. To assess acceptability, we used an adapted version of the Theoretical Framework of Acceptability Questionnaire, which consists of seven domains: affective attitude, burden, perceived effectiveness, ethicality, intervention coherence, opportunity costs, and self-efficacy.^26^ This questionnaire assesses an intervention’s acceptability from the perspective of the participants, and is commonly used to evaluate interventions in various healthcare settings.^27–29^ Additionally, we used a self-developed acceptability questionnaire, which comprised of eight statements with a 4-point Likert scale ranging from “Fully Agree” to “Fully Disagree.” Exemplary statements are “*I would recommend ‘Smoke-Free and MS’ to other people with MS*,” “*I am confident to stay/become smokefree*,” or “*The MS-specific content increased my motivation to quit smoking*” (see also Figure 2).

**Table 1.** Overview of primary and secondary outcomes, covariates, and time of assessment.

Primary outcomes	Item	Time of assessment		
		BL	FU1	FU2
Acceptability (research question 3)	SUS		x	
	TFA Questionnaire (adapted)		x	
	Self-Developed Acceptability Questionnaire		x	
Demand (research question 1)	Recruitment duration	n/a		
	Number of participants approached			
	Number of participants who started the intervention			
	Number of participants who withdrew or were lost to follow-up			
Practicality (research question 2)	Number of participants with complete baseline data	n/a		
	Number of participants who participated in the online group sessions			
	Number of participants with complete follow-up data after 3 weeks			
	Number of participants with complete follow-up data after 12 weeks			
Secondary outcome	Item	BL	FU1	FU2
Potential effectiveness (research question 4)	Smoking status	x	x	x
	Cotinine			x
Other variables	Item	BL	FU1	FU2
Sociodemographic data	Gender	x		
	Age	x		
	Education	x		
	Employment status	x		
	Family status	x		
MS-related data	MS subtype	x		
	Time of diagnosis	x		
	Immune therapy	x		
	PDDS	x		
Quality of life	EQ-5D	x	x	x
Anxiety and depression	PHQ-4	x	x	x
Nicotine dependence	Fagerström Test for Nicotine Dependence	x	x	x
NRT	Use of NRT		x	x

BL: baseline; FU1: follow-up 1 (3 weeks after baseline); FU2: follow-up 2 (12 weeks after baseline); n/a: not applicable; EQ-5D: Euro Quality of Life score; MS: multiple sclerosis; NRT: Nicotine Replacement Therapy; PDDS: Patient Determined Disability Scale; PHQ-4: Patient Health Questionnaire; SUS: System Usability Scale; TFA: Theoretical Framework of Acceptability.

We also assessed the acceptability of our online platform “eRecover.” Here, we used the reliable and widely used System Usability Scale (SUS),^30^ which quantitatively measures perceived usability. It uses 10 statements with a 5-point Likert scale ranging from “strongly disagree” to “strongly agree,” for example: “*I thought the system (eRecover) was easy to use*,” “*I thought there was too much inconsistency in this system (eRecover)*,” or “*I needed to learn a lot of things before I could get going with this system (eRecover)*.” Based on the given answers, a score ranging from 0 to 100 can be computed; a score of 68 is commonly used as a benchmark, with values above this threshold generally interpreted as indicating good acceptability.^31,32^

To test our secondary outcome, potential effectiveness efficacy (research question 4), we used the Fagerström Test of Nicotine Dependence^33^ to measure levels of nicotine addiction, and self-reported smoking status. For the 12-week follow-up, we additionally determined the cotinine level in saliva samples by gas chromatography mass spectrometry to verify the self-reports. The analysis was conducted by the Institute for Occupational and Maritime Medicine, which is part of the Ministry of Justice and Consumer Protection in Hamburg and affiliated with the Medical Faculty of Hamburg University.

In addition to sociodemographic variables, our covariates included three validated instruments: the Patient-Determined-Disease-Steps score (PDDS),^34^ the Euro Quality of Life score (EQ-5D),^35^ and the Patient Health Questionnaire score (PHQ-4).^36^ The PDDS is a self-reported measure of MS-related disability and ranges from 0 to 8, with higher scores indicating greater perceived disability (0 = normal, 1 = mild disability, up to 8 = bedridden).^34^ The EQ-5D assesses health-related quality of life across five dimensions, each rated on a 5-level scale from no problems to extreme problems: mobility, self-care, usual activities, pain/discomfort, and anxiety/depression. Additionally, a visual analog scale from 0 (worst imaginable health) to 100 (best imaginable health) is included. The PHQ-4 is a brief screening tool for mental distress, consisting of two items for anxiety and two for depression, each rated on a 4-point Likert scale (0 = not at all to 3 = nearly every day), resulting in a total score from 0 to 12; higher scores indicate greater symptom severity.

Prior to study start, we defined five progression criteria, which, if fulfilled, support the conduct of a future randomized controlled trial (RCT) to assess efficacy: (1) the successful recruitment of 60 participants within 6 months; (2) a maximum dropout rate of 20% after 12 weeks; (3) a SUS score above 68; (4) a TFA score above 3; (5) a minimum percentage of 80% of study participants who would recommend the program to other pwMS.

### Statistical methods

Data were analyzed descriptively using R-Studio (by Posit PBC) and presented as appropriate. Continuous variables are reported as means with standard deviations, or medians and ranges, as considered appropriate. Categorical variables are reported as absolute frequencies and percentages. Participants with a missing value for smoking status during the follow-ups were treated as smokers.

## Results

### Participant flow and recruitment

#### Demand and practicality

Between November 2024 and May 2025, a total of 130 individuals started the screening on LimeSurvey. Of these, 107 completed the process, and 99 were eligible according to our criteria. Among the eligible participants, 18 did not provide informed consent, and 19 were unable to attend at any of the available course dates. This resulted in 62 enrolled participants.

Out of those enrolled, 14 participants did not start the intervention for unspecified reasons, leaving 48 participants who started the program. On average, these 48 participants took part in 4.1 (median = 5) out of 5 online meetings. Although the initial goal was to recruit 60 pwMS for the intervention, recruitment had to be discontinued after 6 months due to time and budgetary constraints.

In total, 8 group-based courses, each comprising 4–10 participants, were conducted between January and May 2025. The 3-week follow-up questionnaire was completed by 40 (83%), and the 12-week follow-up questionnaire by 37 (73%) participants.

All details, including reasons for drop-outs, are presented in Figure 1.

**Figure 1.**
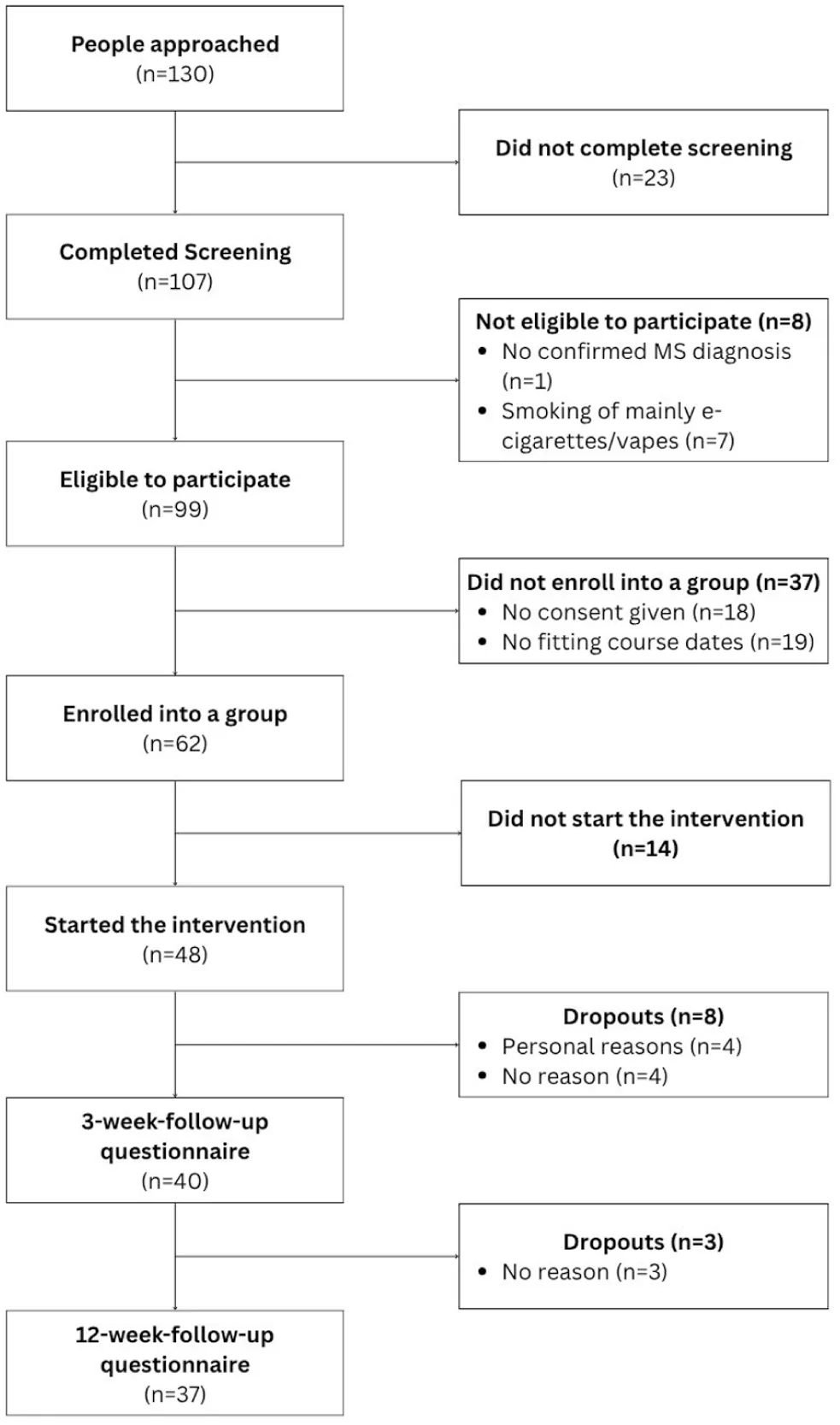
Participant flow diagram.

### Baseline data

Of all participants who started the intervention (*n* = 48), 46 completed the baseline questionnaire. We had 74% (*n* = 34) female participants, and a mean age of 42.6 years in our sample. The average disease duration was 8.6 years, with 58% (*n* = 27) having a relapsing remitting MS. All participants were currently smoking at the start of the intervention, and the mean age for smoking initiation was 16.2 years. We had one participant with low nicotine dependence according to the Fagerström Test, 37.0% (*n* = 17) with a medium, 52.2% (*n* = 24) with a high, and 8.7% (*n* = 4) with a very high nicotine dependence. An overview of all detailed baseline characteristics can be found in Table 2.

**Table 2.** Baseline data of our participants (*n* = 46).

Variable	Total sample (*N* = 46^a^)	
Sex	*n*	%
Male	12	26.0
Female	34	74.0
Age (in years)	Mean (median)	Range
	42.6 (42)	26–68
Education	*n*	%
No school leaving certificate	1	2.2
Secondary school leaving certificate	4	8.7
Intermediate secondary school certificate	13	28.3
High school diploma	28	60.8
Marital status	*n*	%
Single	13	28.2
In a relationship	17	36.9
Married	10	21.8
Separated	5	10.9
Widowed	1	2.2
MS type	*n*	%
Relapsing remitting	27	58.6
Primary progressive	6	13.1
Secondary progressive	7	15.2
Unclear	6	13.1
Disease duration (in years)	Mean (median)	Range
	8.6 (5.4)	0.2–31.7
PDDS (0–8)	Mean (median)	Range
	2.7 (3)	0–8
Ever use of immunotherapy	*n*	%
Yes	31	67.4
No	15	32.6
Current smoking status	*n*	%
Yes	46	100
No	0	0.0
Age of smoking initiation (in years)	Mean (median)	Range
	16.2 (15.5)	12–35
Fagerströms Test of Nicotine Dependence	*n*	%
Low	1	2.1
Medium	17	37.0
High	24	52.2
Very high	4	8.7
EQ-5D-5L dimensions (1–5)	Mean (median)	Range
Mobility	2.1 (2)	1–5
Self-care	1.4 (1)	1–3
Usual activities	2.4 (3)	1–4
Pain/discomfort	2.4 (3)	1–4
Anxiety/depression	2.3 (2)	1–4
Overall score (0–100)	58.6 (60)	20–100
PHQ-4	*n*	%
No or minimal signs of anxiety or depression	22	48.9
Moderate signs of anxiety or depression	18	40.0
Severe signs of anxiety or depression	5	11.1

MS: multiple sclerosis; PDDS: patient-determined disease steps; EQ-5D-5L: European Quality of Life 5 Dimensions 5 Level Version; PHQ: Patient Health Questionnaire.

a For the EQ-5D-5L and the PHQ-4 we had one non-responder, *n* = 45.

### Follow-up data

#### Acceptability

From all participants, 39 filled out the TFA questionnaire after the intervention was completed. All 39 (100%) participants reported that they liked the program, and 84.7% (*n* = 33) said that the participation was not or only a little demanding. In contrast, 12.7% (*n* = 5) agreed, that the participation kept them from engaging in other important activities. However, 89.8% (*n* = 35) agreed or strongly agreed that our program had helped them to change their smoking behavior, and 87.2% (*n* = 34) agreed that the program was suitable or very suitable for them. Based on all given answers, the TFA average score across all five questions was 4.29, with no question having more than 12.7% (*n* = 5) negative answers. All detailed results can be found in Table 3.

**Table 3.** Results from the TFA questionnaire (*n* = 39).

Question	Response option	*N* (%)
Did you like “Smoke-Free and MS”?	Yes, very much	24 (61.5)
	Yes	15 (38.5)
	No opinion	0
	No	0
	Not at all	0
How demanding was participating in “Smoke-Free and MS” for you?	Not demanding at all	15 (38.5)
	A little demanding	18 (46.2)
	No opinion	2 (5.1)
	Moderately demanding	3 (7.7)
	Very demanding	1 (2.5)
“Smoke-Free and MS” helped me change my smoking behavior.	Strongly agree	24 (61.5)
	Agree	11 (28.3)
	No opinion	3 (7.7)
	Disagree	1 (2.5)
	Strongly disagree	0
Participation kept me from engaging in other important activities.”	Strongly disagree	15 (38.5)
	Disagree	15 (38.5)
	No opinion	4 (10.3)
	Agree	5 (12.7)
	Strongly agree	0
How suitable was “Smoke-Free and MS” for you?	Very suitable	14 (35.9)
	Suitable	20 (51.3)
	No opinion	4 (10.3)
	Rather not suitable	1 (2.5)
	Not suitable at all	0
TFA average score	4.29	

MS: multiple sclerosis; TFA: Theoretical Framework of Acceptability Questionnaire.

The average score of the TFA is computed using the answers from the five questions, each reaching from 5 (most positive answer) to 1 (most negative answer); a high average score indicates a high acceptability.

Our self-developed acceptability questionnaire was completed by 39 participants. All 39 (100%) participants agreed or fully agreed that they would recommend the program to other pwMS. All but one (97.4%) stated that they were overall satisfied with the program, and 89.7% (*n* = 35) agreed or fully agreed that they are confident to stay/become smoke-free. As for the MS-specific content, 97.5% (*n* = 38) agreed or fully agreed that the content was relevant for them, and 82.1% (*n* = 32) agreed or fully agreed that the way how the content was presented within the program was appropriate. Finally, 89.7% (*n* = 35) agreed or fully agreed, that the MS-specific content had increased their motivation to stop, that it had been new to them, and that they had used our online platform “eRecover” to rewatch and look up the content after the online sessions as well. All detailed results from the self-developed acceptability questionnaire can be found in Figure 2.

**Figure 2.**
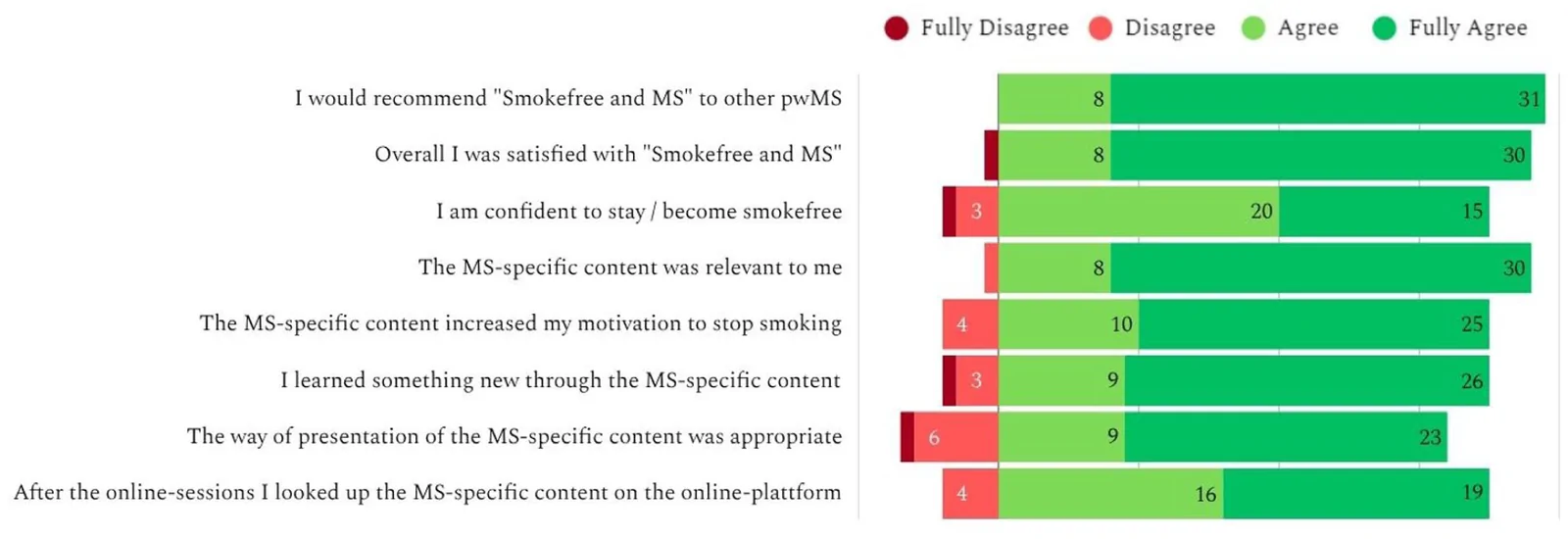
Results for the self-developed acceptability questionnaire.

The SUS questionnaire was completed by 40 participants. Based on all answers, we computed a final SUS score of 75.5, which was above the pre-specified threshold of 68.

#### Potential effectiveness

To assess smoking status, we asked our participants for self-reports at baseline and both follow-ups. The smoking rate at baseline was 100% (*n* = 48). At the first follow-up assessment after the intervention, 3 weeks after baseline, 48% (*n* = 23) reported to be smoke-free, and 35% (*n* = 17) reported to currently smoke. Eight (17%) participants did not answer the follow-up questionnaire and were therefore counted as smokers as well. Taking this into account, we had 48% (*n* = 23) people who quit smoking, and 52% (*n* = 25) who still smoked 3 weeks after baseline.

Twelve weeks after baseline 12 (25%) participants reported being smoke-free, 25 (52%) reported that they were still smoking, and 11 (23%) did not complete the questionnaire. To biochemically verify self-reported abstinence, we sent saliva sample kits between April and August 2025 to all participants who had reported being smoke-free. Of the 12 kits distributed, 8 were returned. Among these, we were able to biochemically verify the smoking status for all samples. So, after 12 weeks, we had a cotinine-verified abstinence rate of 17%. The laboratory results from the cotinine testing can be found in Supplemental File 1.

#### Other variables

For every time point, we assessed nicotine dependence using the Fagerström Test. Baseline scores have already been described above. At first follow-up, out of all participants who reported that they are still smoking (*n* = 17), 11 (65%) participants had a medium, 5 (29%) a high, and 1 (6%) a very high nicotine dependence. At the second follow-up, among all participants who reported that they were still smoking (*n* = 25), 17 participants (68%) had a medium, 6 (24%) a high, and 2 (8%) a very high level of nicotine dependence.

The use of nicotine replacement therapy (NRT) was assessed during both follow-ups. Three weeks after baseline, 8 (20%) out of 40 participants reported to use NRTs. Twelve weeks after baseline, 3 (8%) out of 37 participants reported to use NRTs.

We assessed quality of life (with the EQ-5D), and anxiety and depression (with the PHQ-4) at every time point. Baseline numbers can be seen in Table 2. The overall changes in mean scores for each of the five dimensions of the EQ-5D-5L were small for both follow-ups. The largest change during the first follow-up was observed for the dimension “Usual activities,” and for the second follow-up, for the dimension “Mobility.” All detailed data can be seen in Table 4.

**Table 4.** Follow-up data (3 and 12 weeks) for the EQ-5D-5L and the PHQ-4.

EQ-5D-5L dimensions	First follow-up (3 weeks)			Second follow-up (12 weeks)		
	Mean (SD)	Range	Change to BL (mean)	Mean (SD)	Range	Change to baseline (mean)
Mobility	2.2 (1.2)	1–5	+0.1	2.4 (1.3)	1–5	+0.3
Self-care	1.4 (0.7)	1–3	±0	1.4 (0.7)	1–3	±0
Usual activities	2.2 (0.9)	1–4	−0.2	2.3 (1.1)	1–4	−0.1
Pain/discomfort	2.3 (1.0)	1–4	−0.1	2.5 (1.1)	1–5	+0.1
Anxiety/depression	2.2 (1.1)	1–5	−0.1	2.1 (1.0)	1–5	−0.2
Overall score (0–100)	67.0 (19.8)	25–100	+8.4	64.0 (21.6)	25–97	+5.4
PHQ-4	*n*	%	Change to BL (%)	*n*	%	Change to BL (%)
No or minimal signs of anxiety or depression	23	62.	+13.3	26	70	+21.4
Moderate signs of anxiety or depression	13	35.	−4.9	9	24	−15.7
Severe signs of anxiety or depression	1	3	−8.4	2	6	−5.7

EQ-5D-5L: European Quality of Life 5 Dimensions 5 Level Version; PHQ: Patient Health Questionnaire; BL: baseline; SD: standard deviation.

*N* (EQ-5D-5L, 1st follow-up) = 39; *N* (EQ-5D-5L, 2nd follow-up) = 37; *N* (PHQ-4, 1st follow-up) = 37; *N* (PHQ-4, 2nd follow-up) = 37.

For the PHQ-4, the proportion of participants with no or minimal signs of anxiety or depression increased at both the first and second follow-up compared to baseline, while the proportion of participants with moderate or severe signs of anxiety and depression decreased. At baseline, only 49% (*n* = 22) reported no or minimal signs. This proportion rose to 62% (*n* = 23) at the first follow-up, and further to 70% (*n* = 26) at the second follow-up. Detailed numbers can be found in Table 4.

### Drop-outs

Of the 11 participants who dropped out, 4 provided direct feedback, citing personal reasons (e.g., time constraints or health-related issues) for their discontinuation. Additionally, to assess potential attrition bias, we compared the baseline characteristics of all non-completers with those who completed the intervention (Table 5). Variables like PDDS, disease duration, sex, age, and nicotine dependence, are similar in both groups. However, none of the non-completers were single (compared to 35% for the completers), and a higher proportion of non-completers had a high school diploma (78% vs 57%) compared to lower educational categories.

**Table 5.** Comparison of baseline characteristics between completers and non-completers of the intervention.

Variable	Completers (*n* = 37)		Non-completers (*n* = 9^a^)	
	*n*	%	*n*	%
Sex				
Male	10	27	2	22
Female	27	73	7	78
Education				
No school leaving certificate	1	3	0	0
Secondary school leaving certificate	3	8	1	11
Intermediate secondary school certificate	12	32	1	11
High school diploma	21	57	7	78
Marital status				
Single	13	35	0	0
In a relationship	13	35	4	45
Married	7	19	3	33
Separated	4	11	1	11
Widowed	0	0	1	11
MS type				
Relapsing remitting	20	54	7	78
Primary progressive	6	16	0	0
Secondary progressive	7	19	0	0
Unclear	4	11	2	22
	Mean	Median	Mean	Median
Age (in years)	42.6	42	42.3	44
Disease duration (in years)	8.2	5	11	8
PDDS	2.8	3	2.6	3
Fagerström-score	5.5	6	5.6	6

MS: multiple sclerosis; PDDS: patient-determined disease steps.

a We had a total of *n* = 11 non-completers, but only complete available data for 9 of them.

## Discussion

This study aimed to assess the feasibility, acceptability, and potential effectiveness of a newly developed smoking cessation program tailored for pwMS. Our findings suggest that the intervention was well accepted and that its implementation is feasible, if the observed limitations are addressed.

The intervention demonstrated high levels of acceptability. The gathered feedback from our participants indicated a high level of satisfaction, particularly regarding the MS-specific content. Many participants described the information as new, relevant, and motivating, which likely contributed to the overall engagement. Importantly, 100% of those who completed the evaluation stated they would recommend the program to others. In addition, results from the SUS suggest that the technical implementation was positively received.

While the study did not meet the originally intended sample size of 60 participants, we still consider the final enrollment of 48 to be satisfactory, especially in light of the narrow target group and logistical demands of the program. Recruitment was constrained by a limited project time frame and budget, which prevented us from offering additional course slots. Furthermore, some individuals who expressed initial interest were unable to participate due to scheduling conflicts. The program format, consisting of five 90-min group sessions, may have posed a barrier for some, despite efforts to provide flexible scheduling options, including morning, evening, and weekend sessions. Nevertheless, the high number of individuals who completed the initial screening reflects a clear demand for such a tailored intervention. This is particularly noteworthy given that a previous MS-specific smoking cessation study failed due to insufficient enrollment.^37^ Program adherence was high, with over 80% of participants completing all sessions. Of the 11 dropouts, 4 were due to personal or health-related reasons rather than dissatisfaction with the program itself. All other dropouts did not specify a reason for discontinuation.

In our feasibility study, self-reported abstinence rates were 48% at 3 weeks and 25% at 12 weeks. Although we expect some decline over time—as is commonly observed^38^—these rates suggest that our intervention may support abstinence well beyond the levels seen without structured support.^39^ Unfortunately, there are no data available known to us reporting quit rates specifically of pwMS of other smoking cessation interventions. Reported abstinence rates for smoking cessation in other studies vary depending on the type of intervention or observed follow-up duration. A study comparing two different web-based programs yielded 21%–23% abstinence after 1 year,^40^ while some pharmacotherapy studies report 13%–23% after 52 weeks,^41^ and a systematic review and meta-analysis of various smoking cessation intervention types reported an average abstinence rate of 26%.^42^ In contrast, unaided quit attempts are generally associated with much lower success rate of around 3%–5% after 1 year.^39^ Also, since our 12-week results are not comparable with 12-month abstinence rates of other studies, they can serve only as a preliminary signal of efficacy and as a predictor of 12-month abstinence. The primary focus of this study was to establish feasibility and acceptability of our intervention. Definitive long-term quit rates (e.g., at 12 months) should be the primary endpoint of subsequent and adequately powered confirmatory RCTs.

Three of our five pre-specified progression criteria were met. In particular, we exceeded the SUS and TFA benchmark, and obtained a 100% recommendation rate among participants who completed the survey. We did not reach the recruitment goal of 60 participants within the specified timeframe and missed our predefined dropout threshold of 80% at the 12-week follow-up. To address these missed criteria, recruitment in future trials should increase scheduling flexibility and expanding outreach through a broader range of communication channels. Although such strategies may require additional financial resources, they could substantially enhance study visibility and accessibility. Moreover, offering more session times may also increase adherence, especially for participants who indicated having to balance other important responsibilities.

The systematic tailoring of smoking cessation interventions for specific chronic conditions is an evolving field, recognizing that generic programs often fail to address disease-specific barriers. Our approach of adapting an evidence-based program is conceptually similar to interventions developed for other chronic populations, such as the “Positively Smoke-Free” (PSF) program for people living with HIV^43^ or the SCIMITAR+ intervention for individuals with severe mental illness,^44^ which resulted in quit rates comparable to our and aforementioned studies (PFS: 19% after 3 months; SCIMITAR+: 14% after 6 months). Similar to PSF, which addresses HIV-specific stressors like stigma and interaction with antiretroviral therapy, our intervention targets unique MS-specific concerns like, for instance, the fear that nicotine withdrawal could trigger an MS relapse, or the interaction of smoking with MS-medication—factors that would not be discussed in generic smoking cessation programs. The primary advantage of such a tailored approach is increased participant relevance and identification, which is reflected in our recommendation rates and adherence rates. Additionally, in our case, by utilizing an online format, we also address MS-specific mobility and fatigue issues which might influence program adherence for this specific target group.

### Strengths and limitations

This study has several strengths. It is the first to evaluate a tailored group-based smoking cessation intervention in a MS-specific setting. We demonstrated high levels of acceptability, with a good program adherence and high willingness to recommend it to other pwMS. The use of established evaluation tools (SUS, TFA) and the biochemical validation in addition to the self-reports adds to the methodological robustness. This study also has some limitations. First, smoking status was primarily self-reported, although we used biochemical verification for the second follow-up. Second, the relatively intensive group format may not be suitable for all individuals, especially those with time constrains, or cognitive difficulties. Third, recruitment relied on low-cost dissemination methods due to budget limitations, which may have affected reach and diversity of the group. Moreover, a potential selection bias must be considered, as adequate German language skills were required for participation. Even though German language skills were no official eligibility criteria this may have excluded individuals with lower educational levels or limited language skills. The relatively high proportion of participants with higher educational attainment further suggest that the sample may not fully reflect the broader population of pwMS who smoke. Also, long-term outcomes were not assessed beyond 12 weeks, leaving the sustainability of the smoking cessation uncertain beyond that time point. Finally, we found some differences in the baseline characteristics regarding marital status and education between completers and non-completers of the intervention. However, given that the primary clinical and smoking-related indicators were balanced, and while also considering the small sample size underlying this comparison, we judge that the observed differences are unlikely to have introduced a systematic selection bias regarding our outcomes.

## Conclusion

Our MS-specific smoking cessation intervention was deemed feasible and acceptable and showed preliminary signs of potential effectiveness in aiding smoking cessation. Despite not fulfilling all of our progression criteria regarding recruitment and drop-out, we are optimistic that by addressing observed limitations in the planning and implementation of a future efficacy trial can be realistic.

Future studies should consider assessing participants’ readiness to change, which may have provided valuable context for interpreting results, since the trainers had the impression that readiness could vary greatly between participants. Furthermore, future studies should investigate the influence of group dynamics on individual outcomes, as we did not systematically assess this potentially influencing factor.

Beyond the implications for pwMS, our findings suggest that disease-specific tailoring may be a promising strategy for increasing the relevance and acceptability of smoking cessation interventions more broadly. The high acceptability and positive reception of the MS-specific content in our study, as well as examples from smoking cessation interventions designed for people with HIV or severe mental illness,^43,44^ indicate that similar approaches could be beneficial for other populations with chronic or neurological conditions. Future research should explore the potential of adapting diagnosis-specific cessation programs for other patient groups in order to maximize potential reach and impact.

## Supplemental Material

sj-docx-1-taj-10.1177_27558428261444225 – Supplemental material for A tailored smoking cessation intervention for people with multiple sclerosis: Results of a single-arm feasibility study Supplemental material, sj-docx-1-taj-10.1177_27558428261444225 for A tailored smoking cessation intervention for people with multiple sclerosis: Results of a single-arm feasibility study by Alex M. Keller, Daniel Kotz, Claudia H. Marck, Martin Winter, Chris M. Friemel, Christoph Heesen and Karin Riemann-Lorenz in Sage Open Chronic Disease

sj-pdf-2-taj-10.1177_27558428261444225 – Supplemental material for A tailored smoking cessation intervention for people with multiple sclerosis: Results of a single-arm feasibility study Supplemental material, sj-pdf-2-taj-10.1177_27558428261444225 for A tailored smoking cessation intervention for people with multiple sclerosis: Results of a single-arm feasibility study by Alex M. Keller, Daniel Kotz, Claudia H. Marck, Martin Winter, Chris M. Friemel, Christoph Heesen and Karin Riemann-Lorenz in Sage Open Chronic Disease

## Data Availability

The datasets used and analyzed during the current study are available from the corresponding author* on reasonable request.
