# Supplementary material for: A tailored smoking cessation intervention for people with multiple sclerosis: Results of a single-arm feasibility study
Source: Sage Open Chronic Dis. 2026 May 16;17:27558428261444225. doi: 10.1177/27558428261444225 (PMC13768618; doi:10.1177/27558428261444225)
Supplement: Supplementary file 2 [file sj-docx-1-taj-10.1177_27558428261444225.docx]

**Results from the Cotinine Testing**

| **#** | **Cotinine [µg/L]** |
| --- | --- |
| 01 | <LOQ |
| 02 | <LOQ |
| 03 | <LOQ |
| 04 | 1,02* |
| 05 | <LOQ |
| 06 | <LOQ |
| 07 | <LOQ |
| 08 | <LOQ |
| LOQ = Limit of Quantification = 0.8µg/L  Cut-off = 15 µg/L  *no clear identification, because the quantitator/qualifier ratio is outside the tolerance | |

Explanation: In seven out of eight samples we could not detect cotinine. In the one other sample the cotinine concentration was below the cut-off of 15 µg/L (1) , indicating nicotine abstinence for a prolonged period of time.

1.	Toraño JS, van Kan HJM. Simultaneous determination of the tobacco smoke uptake parameters nicotine, cotinine and thiocyanate in urine, saliva and hair, using gas chromatography-mass spectrometry for characterisation of smoking status of recently exposed subjects. Analyst. 2003 Jul;128(7):838–43.
